# Impact of sensory integration in modulating neuronal oscillation bands and antagonist vibratory response during co-vibration

**DOI:** 10.3389/fnhum.2026.1749961

**Published:** 2026-07-29

**Authors:** Khin Win Thu, Kenya Tanamachi, Megumi Okawada, Wataru Kuwahara, Fuminari Kaneko

**Affiliations:** 1 Department of Physical Therapy, Graduate School of Human Health Sciences, Tokyo Metropolitan University, Tokyo, Japan; 2 Department of Rehabilitation Medicine, Keio University School of Medicine, Tokyo, Japan

**Keywords:** antagonist vibratory response, event-related desynchronization, kinesthetic illusion, neuronal oscillations, tendon vibration

## Abstract

**Introduction:**

Kinesthetic illusion (KI) induced by tendon vibration (VIB) is known for activation in sensorimotor areas and antagonist vibratory response (AVR) of the antagonist muscle. We hypothesize that coupling between cortical activation and corresponding AVR activity occurs during KI induced by VIB. This study aimed to explore the neurophysiological features of KI induced by VIB associated with brain activity and muscle activity.

**Methods:**

Two experimental sets, without-hand-cover (wo) and with-hand-cover (w), included three co-vibration frequency patterns applied to the flexor carpi radialis and extensor carpi radialis muscles: 80:80 Hz, 115:45 Hz, and 70:0 Hz, resulting in six conditions.

**Results:**

AVR-electromyography (EMG) activity differed significantly between w-70:0 vs. wo-70:0 and between w-70:0 vs. w-80:80 (adjusted *p* < 0.05). Event-related desynchronization (ERD) of electroencephalography did not differ significantly between conditions. KI strength was significantly higher in both w-115:45 and w-70:0 conditions than in w-80:80 conditions (adjusted *p* < 0.01). Spearman’s correlation for w-conditions (w-115:45 and w-70:0) revealed a significant correlation between both *α*-and *γ*-ERD at C3 with AVR-EMG activity (*ρ* = 0.37, *p* < 0.05) and a significant negative correlation between *γ*-ERD with KI at CP3 (*ρ* = −0.36, *p* = 0.049) and P3 (*ρ* = −0.40, *p* = 0.027). No similar correlation was observed for wo-conditions.

**Discussion:**

These significant correlations emerged under distinct KI conditions, suggesting condition-and region-specific cortical engagement.

## 1 Introduction

Neuronal oscillation bands of event-related desynchronization (ERD) are observed during tendon vibration (VIB), reflecting sensorimotor processing without actual movement (Casini et al., 2006; Adham et al., 2024). These oscillatory patterns resemble actual movement execution, confirming that vibration modulates cortical rhythms by creating a kinesthetic illusion (KI), which the brain interprets as real movement (Roll and Vedel, 1982; Romaiguère et al., 2003). This occurs because VIB excites Ia afferents from the muscle spindles, which relay precise proprioceptive information regarding changes in muscle length to the brain (Goodwin et al., 1972; Roll and Vedel, 1982). Consequently, this creates a mismatch between false and actual stretch signals. To reconcile this, the brain generates KI, producing a vivid perception of movement despite the absence of actual movement (Roll and Vedel, 1982; Proske and Gandevia, 2018).

Accordingly, KI induced by VIB recruits the associative sensorimotor cortical network (Romaiguère et al., 2003) and can also be an alternative form of motor imagery. It is especially useful in cases of limitations in performing motor imagery in physiotherapy rehabilitation settings (Kito et al., 2006). In addition, KI induced by VIB is associated with muscle responses from the antagonist muscles of the vibrated muscle, known as antagonist vibratory responses (AVRs) (Gilhodes et al., 1986; Calvin-Figuière et al., 1999). Previous studies vary in methodologies, using different VIB frequency ranges (e.g., 40, 60, 100 Hz) and stimulation patterns—ranging from single VIB on one muscle side to co-VIB on two muscle sides—while also incorporating different vision conditions where the vibrated arm is either visible or hidden from view. Approximately 70–80 Hz frequency for single VIB or co-VIB, frequency differences between two muscle sides in the absence of visual feedback condition, can induce sufficient observability of its effect (Romaiguère et al., 2003; Taylor et al., 2017; Proske and Gandevia, 2018; Le Franc et al., 2020). Consequently, the degree of KI can vary, ranging from no illusion to a strong KI, depending on the VIB pattern and frequency, with corresponding activation differences in the sensorimotor and parietal cortical regions (Romaiguère et al., 2003; Naito et al., 2016).

Neuronal oscillation plays a fundamental role in regulating brain function, and synchronization of its specific neuronal frequency bands’ activity reflects distinct cognitive processes. Neuronal oscillations in distinct frequency bands, the alpha-band (*α*), beta-band (*β*), and gamma-band (*γ*), are associated with specific neural functions: alpha rhythms regulate sensory inhibition by suppressing irrelevant or distracting sensory inputs; beta mediate motor planning, motor execution to reflect the maintenance of sensorimotor states, while gamma support higher order cognitive processes through sensory gating, which involves filtering relevant sensory inputs, and contribute to perceptual binding and conscious awareness (Jensen and Mazaheri, 2010; Haegens et al., 2011; Barone and Rossiter, 2021; Guan et al., 2022). Moreover, these specific neuronal oscillation bands can be impaired by brain-related diseases, each associated with characteristic functional impairments. For example, unbalanced alpha activity may contribute to sensory neglect, disrupted beta oscillations to motor dysfunction, and attenuated gamma synchrony to cognitive deficits. Therefore, these impairments influence motor recovery outcomes during rehabilitation, depending on individual requirements and the extent of their impairment (Başar et al., 2013; Storch et al., 2021; Leonardi et al., 2022; Harquel et al., 2025; Samantzis et al., 2025).

Many previous studies have been conducted to induce neuronal oscillation activity by implementing different proprioceptive stimulations: visual stimulation (VIS), VIB, and combined VIS with electrical stimulation, for rehabilitation purposes (Naito et al., 2016; Kaneko et al., 2017; Shibata and Kaneko, 2019; Okawada et al., 2020; Snyder et al., 2023). Additionally, researchers have highlighted the distinct roles of neuronal oscillation bands, including *α*, *β*, and *γ*, in different sensory integration processes, particularly under tactile stimulation and VIS, in different cortical brain areas (Ryun et al., 2017; Riddle et al., 2019). The α band could be an objective parameter representing KI of VIS; a VIS study conducted post-stroke revealed a significant increase in the β band ERD after 20 min of VIS (Shibata AND Kaneko, 2019; Okawada et al., 2020). However, whether KI induced by VIB with a different VIB pattern is represented by the same frequency band remains to be elucidated.

Regarding VIB followed by KI, previous studies have explored the influence of physiological aspects, such as the enhancement or suppressive effect of vision and different vibration patterns on brain and muscle activities as an outcome of interest (Calvin-Figuière et al., 2000; Romaiguère et al., 2003; Forner-Cordero et al., 2008; Proske and Gandevia, 2018; Lauzier et al., 2023). However, the association among these variables, especially the correlation between specific neuronal oscillation frequency bands of ERD coupling with AVR in responses to KI of VIB, remains unexplored. Therefore, this study aimed to examine how multisensory integration affects the online processes of cortical activation via ERD in specific frequency bands and the peripheral activities during co-vibration in healthy participants, focusing on frequency patterns and visual input of the vibrated forearm.

The purpose of this study was to elucidate how sensory integration mechanisms influence cortical-peripheral activity as a neurophysiological parameter with the appearance of KI induced by VIB. This clarification could provide fundamental insights for the development of evidence-based neurorehabilitation protocols, potentially guiding the refinement of multisensory stimulation to enhance motor recovery in patients with sensory-motor impairment, including post-stroke. We hypothesized that ERD modulation in *α*-, *β*-, and *γ*-bands over sensorimotor (C3/CP3) and parietal (P3) regions would differ depending on vibration condition and visual context (hidden limb vs. visible limb), and that stronger KI states would be associated with greater cortico-peripheral coupling between ERD and AVR-EMG activity.

## 2 Methods

### 2.1 Participants

We recruited 15 healthy right-handed participants (six men and nine women) with a mean age of 26 years (± 3.7 years) and a body mass index of 22.8 kg/m^2^ (± 3.5 kg/m^2^) from Tokyo Metropolitan University. Participants who had neurological diseases or were undergoing neuromodulatory or psychiatric-related treatments were excluded from our study.

This research followed the principles of the Declaration of Helsinki. Informed consent was obtained from all participants before the experiment, including consent for the publication of identifying information/images in an open-access publication.

### 2.2 Instruments and measures

#### 2.2.1 Electroencephalography and electromyography

Electroencephalography (EEG) signals were acquired using the Geodesic EEG system 400 MR (Electrical Geodesics Inc., Eugene, OR, USA) and Net Station v5.2 recording software at a sampling rate of 1,000 Hz (Saur et al., 1996). A 128-channel, high-impedance HydroCel Geodesic Sensor Net was used with an electrode impedance of 50 kΩ. The EEG bandpass filter was set to 0.5–200 Hz with a 50 Hz notch filter. Moreover, reference electrodes were averaged across all electrodes.

EMG signals were recorded using the Delsys Trigno surface EMG (sEMG) system (USA). To measure muscle activity, two electrodes were placed at one-third along the length of the right flexor carpi radialis (FCR) and extensor carpi radialis (ECR) muscles. The peak-to-peak of the noise at rest was <20 μV. The EMG bandpass filter was set to 5–500 Hz with a 50 Hz notch filter. Data sampling was set at 2,000 Hz. We did not notch out the stimulation frequencies because reflex-related EMG responses are broadband and not strictly phase-locked to the vibration waveform; removing these frequencies could eliminate physiological components of AVR. Instead, artifact control relied on spatial separation between vibration sources and recording electrodes, baseline recordings during vibration-free periods, and muscle-specific activation patterns across conditions.

#### 2.2.2 7-point Likert scale

Kinesthetic senses, including the sense of body position and movement, were used to assess vibration-induced kinesthetic illusion by administering questionnaires (Proske and Gandevia, 2018). The 7-point Likert scale was used to assess the degree of KI, sense of body ownership, and sense of agency (Okawada et al., 2020). Based on previously established methodologies (Proske and Gandevia, 2018; Okawada et al., 2020), the following questionnaire was used in this study to assess the sense of kinesthetic perception and agency during co-vibration:

1. Did you feel an illusory movement of your hand during stimulation?
2. Did you feel an extension direction of your hand during stimulation?
3. Did you feel a flexion direction of your hand during stimulation?
4. Did you feel as if you were controlling your hand during stimulation?

Responses were rated on a 7-point Likert scale as strongly agree, agree, somewhat agree, neither agree nor disagree, somewhat disagree, disagree, and strongly disagree as +3, +2, +1, 0, −1, −2, −3, respectively.

### 2.3 Procedure

Participants were instructed to sit in a chair with their forearms resting on the forearm support on the experiment table. The chair height was adjusted to maintain the resting position. Subsequently, participants were asked to wear earplugs to reduce external and vibration noise. The researcher measured the nasion-to-inion and tragus-to-tragus distance of the participants’ heads to mark a vertex for the precise Cz location of the 128-channel EEG net and the circumference of the participants’ heads to determine the size of the EEG net. The EEG net was soaked in a conductive solution to reduce impedance between the scalp and the electrode.

During the pre-soaking EEG net stage, the skin was prepared using an alcohol swab and a skin-prepping gel to reduce skin impedance. sEMG electrodes were attached at one-third along the length of the right FCR and ECR muscles. Participants were asked to hold the movable manual portable pole on each side without applying force and maintain the wrist joint at 0^o^ to adjust both forearms to the same resting position. To minimize vibration artifacts, sEMG electrodes were placed over muscle bellies to maintain spatially separation from the vibration source. Two vibrators were attached to the distal part of the right FCR and ECR muscles, just above the wrist joint, and adjusted to induce KI accordingly. These vibrators were secured using elastic Velcro straps. To prevent displacement, the attachment location was marked on the skin; and the same investigator performed all attachments using consistent strap tension to ensure stable contact force. This setup ensured a fixed displacement (amplitude) of 1 mm (Adham et al., 2024). Although contact force was not instrumentally quantified, standardized attachment procedures and consistent investigator handling were used to minimize inter-participant variability.

Subsequently, saline solution was sprayed on the participants’ hair to reduce impedance before the EEG net was positioned to align the vertex and Cz electrode with accurate placement. The electrode impedance was checked and adjusted between the scalp and electrodes with saline solution, if required. Electrode impedance was checked before each co-vibration condition.

Participants’ KIs were assessed before each co-vibration condition. The participants were asked to close their eyes and report their sense of illusion during the co-vibration and single vibration conditions. Subsequently, we recorded 30-s resting ERD and EMG activities when the participants were relaxed, with no stimulation (0 Hz) applied. ERD and EMG activities were measured simultaneously for the resting state and during co-vibration conditions. During each EEG recording, participants were instructed to remain still, minimize blinking, and wear earplugs to reduce noise artifacts on EEG data.

**Figure 1:**
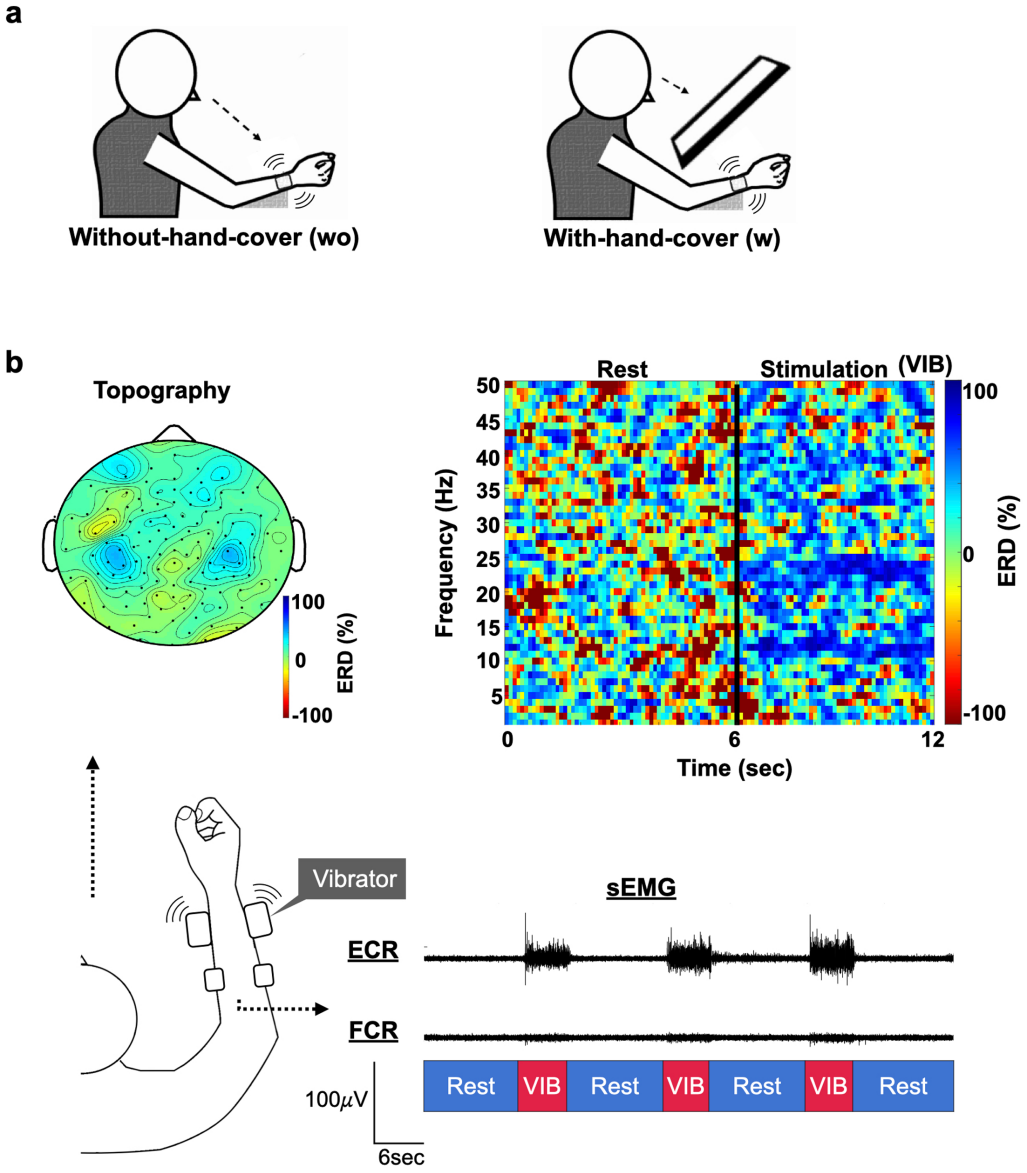
Experimental protocol **(a)** without-hand-cover (wo): participant looked directly at the vibrated forearm, and with-hand-cover (w): the participant’s vibrated arm was covered with a black screen and was asked to focus on the designated point of the screen. **(b)** Data flow schematic of vibration responses: Event-related desynchronization (ERD) activities of topography, time-frequency plot of power spectral density, and surface electromyography (sEMG) activities. VIB, tendon vibration; ECR, extensor carpi radialis; FCR, flexor carpi radialis.

We delivered the co-vibration conditions in a fixed block sequence to maintain a stable sensory state and minimize potential carry-over effects. Participants first completed a set of without-hand-cover (wo) conditions, followed by a set of with-hand-cover (w) conditions (Figure 1a). This non-randomized, sequential approach was adopted to ensure that the potentially higher KI elicited during w-set did not interference with the perception of the wo-set thereby preventing residual kinesthetic effects from biasing the results. Immediately following each stimulation trial, participants were instructed to report their perceived illusion (flexion or extension) with their left wrist joint movement. At the end of each co-vibration condition, participants were instructed to identify their feelings using the 7-point Likert scale questionnaire.

### 2.4 Tendon vibration

We used two vibrators (TECHNO CONCEPT, VB115) and investigated six co-vibration conditions across two experimental sets: wo-set and w-set (Figure 1a). The co-vibration conditions were based on three frequency patterns for FCR and ECR:

1. 80:80 Hz (total frequency: 160 Hz, 0 Hz frequency difference)
2. 115:45 Hz (total frequency: 160 Hz, 70 Hz frequency difference)
3. 70:0 Hz (total frequency: 70 Hz, 70 Hz frequency difference).

These frequency patterns were selected to compare the effects of frequency differences from total sensory load. We setup 115:45 Hz pattern to match total frequency of the 80:80 Hz condition (160 Hz), while maintaining a 70 Hz difference known to elicit kinesthetic illusion. The upper frequency of 115 Hz reflects the operational limit of the vibration apparatus. The 70:0 Hz condition provided an additional comparison with the same 70 Hz difference while reducing the total sensory load. These conditions were labeled and delivered in a fixed sequence as wo-80:80, wo-115:45, wo-70:0 for wo-set, followed by w-80:80, w-115:45, w-70:0 for w-set. Therefore, six co-vibration conditions were included.

In the wo-set (visible limb context), participants were asked to look directly at their vibrated arm. In contrast, in the w-set (hidden limb context), participants’ forearms were blocked from their view by a black screen, and they were instructed to focus only on the screen (Figure 1a). Each stimulation trial comprised three repetitions of 6 s of stimulation followed by 12 s of rest period. This interval was specifically maintained to minimize the risk of sensory adaptation to repeated vibration. Each co-vibration condition involved three trials, which were delivered in a fixed sequence described above.

### 2.5 Data analyses

ERD and EMG analyses were performed using a custom-written code in MATLAB (MATLAB R2023a; The MathWorks Inc., Natick, MA, USA). For EEG signals, we analyzed data from six electrode sites (C3, CP3, P3, C4, CP4, and P4) identified as regions of interest (ROIs) following the international 10–10 electrode positioning system, as our study targeted to explore KI induced by VIB. These electrodes overlay the primary sensorimotor cortex (C3/C4), somatosensory association areas (CP3/CP4), and posterior parietal cortex (P3/P4), which are directly implicated in KI-related proprioceptive integration; therefore, cortical source localization was not performed. For the time-frequency analysis of the EEG, we used the Fast Fourier Transform with a 1-s window length, performed every 100 ms. Then, for the *α* (8–13 Hz), *β* (14–29 Hz), and low *γ* (30–49 Hz) bands analyses, we used the following equation (Shibata and Kaneko, 2019):

ERD(f,t)={[R(f)−S(f,t)]/R(f)}×100%

**Figure 2:**
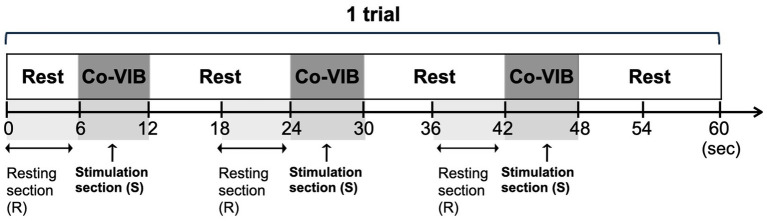
Data analysis interval. The resting section was 6 s before the stimulation time. The stimulation time was 6 s of the vibration time of all trials.

Where *R*(*f*) is the power spectrum of the EEG at the same frequency of the resting section, which was 6 s before the stimulation time of all trials; *S*(*f*,*t*) is the power spectrum of the EEG at a certain frequency and stimulation time, which was 6 s from the stimulation time of all trials (Figure 2). Mean ERD values were first calculated for each frequency within the target frequency band. We identified the frequency showing the highest mean ERD and averaged the values at this frequency plus at 1 Hz before and after the frequency (total 3 Hz). This analysis account for inter-individual differences in the frequency of maximal desynchronization (Shibata and Kaneko, 2019; Okawada et al., 2020). During preprocessing, individual repetitions were visually inspected for ocular and other prominent transient artifacts. Repetitions containing blink-like or large-amplitude artifacts were excluded from subsequent analysis. Artifact handling was performed through visual inspection and exclusion of contaminated repetitions. Independent component analysis, automated channel rejection, and channel interpolation were not performed.

Moreover, an additional exploratory spectral assessment was performed to evaluate potential vibration-related mechanical contamination. EEG signals from the predefined electrodes channels were segmented into 6-s rest and 6-s vibration epochs. Each epoch was linearly detrended, and after which one-sided power spectral density (PSD) was computed using FFT. The resulting spectra from the rest and vibration periods were visually compared over the 0-50 Hz frequency range to assess possible vibration-related spectral features.

For EMG analysis, EMG amplitude was quantified using root mean square (RMS) amplitude, which is based on signal squaring. RMS amplitude was calculated separately for the FCR and ECR using a 1-s analysis window and the mean RMS amplitude was calculated during each 6-s stimulation period. EMG amplitudes were analyzed in their original units and were not normalized to maximal voluntary contraction or baseline activity. In addition, mean AVR-EMG was calculated as the difference between the mean ECR and FCR RMS amplitudes (ECR-FCR). A positive AVR-EMG value indicates ECR greater than FCR activity.

### 2.6 Statistical analyses

All statistical analyses were conducted using IBM SPSS Version 27.0 (IBM, USA). Descriptive statistics were used to describe participants’ demographics. The Shapiro–Wilk test confirmed that the data did not follow a normal distribution. The Friedman test was used to analyze all ERD bands (*α*, *β*, and *γ*) at ROI sites (C3, CP3, P3, C4, CP4, and P4), AVR-EMG activity, and 7-point Likert scale responses related to the KI of all conditions. *Post hoc* pairwise comparisons were performed using Wilcoxon signed-rank test with Bonferroni adjustment. Kendall’s W was reported as the effect size for the Friedman test.

Spearman’s correlation coefficient (*ρ*) was used to explore the relationship between cortical (ERD) and peripheral muscle activities (AVR-EMG activity) and examine how sensory integration influences both pathways. Statistical significance was set at *p* < 0.05. An *a priori* power analysis (G*Power 3.1.9.7) indicated that a sample size of *N* = 19 would be required to detect medium effect sizes (*f* = 0.25) for within-subject comparisons. After applying predefined inclusion and data-quality criteria, the final analyzable sample consisted of 15 participants. A *post-hoc* sensitivity analysis indicated that, with *N* = 15 and six repeated measurements, the study was powered to detect effects of approximately *f* = 0.28. Therefore, the present sample was sensitive to medium-to-large effects (*f* ≥ 0.28), whereas smaller effects may not have been detectable.

Non-normally distributed data are presented as median (interquartile range, IQR) throughout all figures and tables and visualized using box plots for the figures in which dots beyond whiskers are outliers.

## 3 Results

### 3.1 7-point Likert scale for a sense of kinesthetic perception and agency

**Figure 3:**
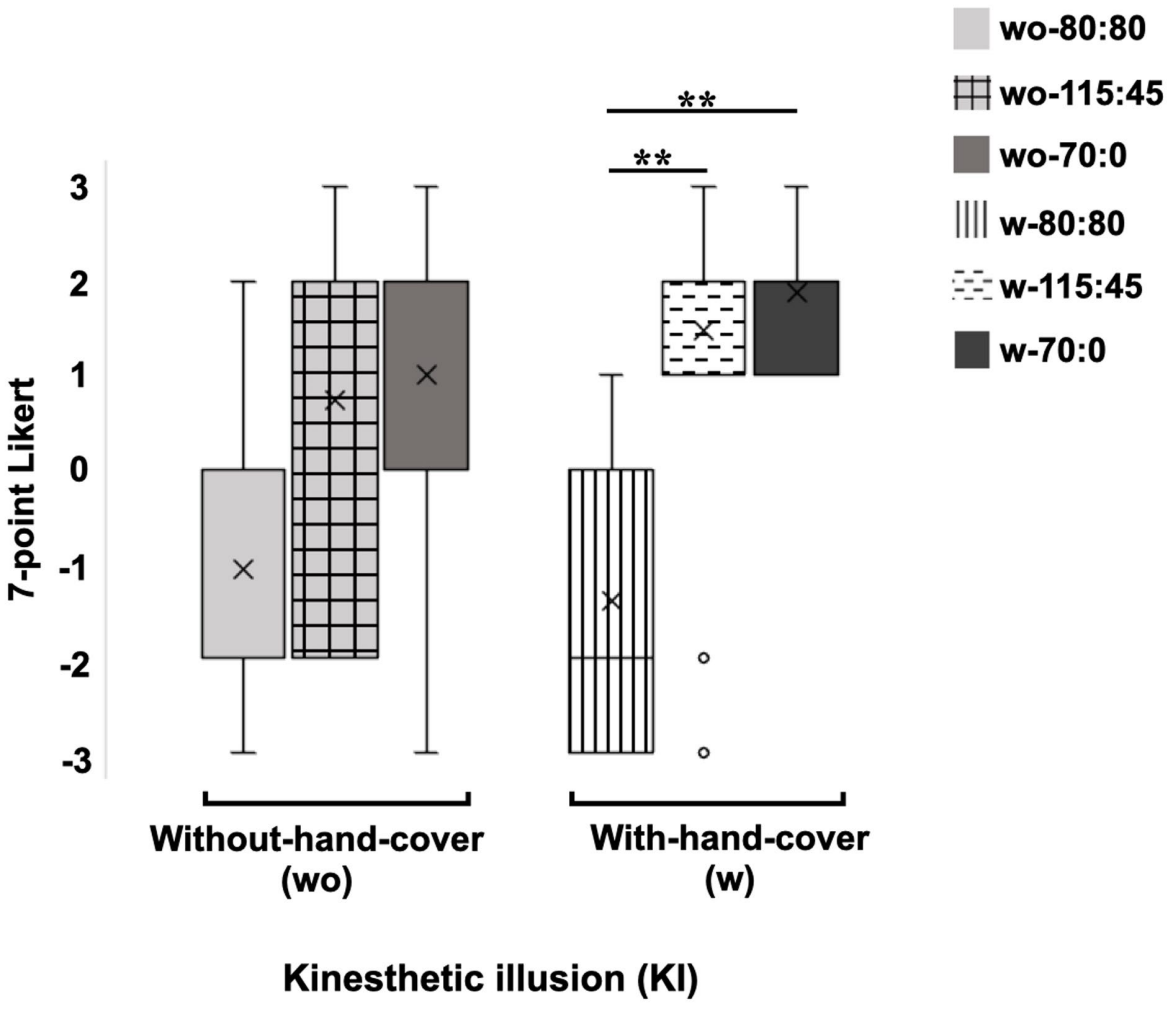
*Post hoc* test comparison of the 7-point Likert scale of KI for all conditions. **Adjusted *p* < 0.01. KI, kinesthetic illusion.

Friedman’s test revealed significant differences between the conditions for KI (*p* < 0.001) and extension direction (Ext) of KI (*p* = 0.000); however, no significant differences were observed for flexion direction (Flex) of KI (*p* = 0.61) and sense of agency (*p* = 0.44). *Post hoc* pairwise comparison with Bonferroni correction showed:

1. For KI: significant differences between w-80:80 vs. w-115:45 (adjusted *p* = 0.002) and w-80:80 vs. w-70:0 (adjusted *p* = 0.001) (Figure 3).
2. For Ext of KI: significant differences between w-80:80 vs. w-115:45 (adjusted *p* = 0.003) and w-80:80 vs. w-70:0 (adjusted *p* = 0.002) and between wo-80:80 vs. wo-70:0 of the wo-condition (adjusted *p* = 0.027).

Furthermore, as shown in Table 1 and Figure 3, w-conditions (w-115:45 and w-70:0) induced higher KI and Ext of KI than wo-conditions (wo-115:45 and wo-70:0). In contrast, neither wo-80:80 nor w-80:80 could induce meaningful KI (Table 1). Therefore, w-115:45 and w-70:0 could be representative conditions for distinct KI states.

**Table 1:** Median (interquartile, IQR) scores for 7-point Likert scale for KI, Ext of KI, Flex of KI, and kinesthetic sense of agency, and AVR-EMG activity for all conditions.

Conditions	Median (IQR)				
	KI	Ext of KI	Flex of KI	Agency	AVR-EMG (μV)
wo-80:80	−2 (−2, 0)	−2 (−2, 0)	−2 (−3, −1)	−2 (−3, −2)	−0.10 (−0.27, 0.42)
wo-115:45	2 (−2, 2)	2 (−1, 2)	−2 (−3, −2)	−2 (−3, −2)	0.26 (−0.89, 1.24)
wo-70:0	2 (0, 2)	2 (1, 2)	−3 (−3, −2)	−3 (−3, −2)	0.14 (0.14, 0.79)
w-80:80	−2 (−3, 0)	−2 (−3, 0)	−2 (−3, −2)	−2 (−3, −2)	−0.02 (−0.27, 0.84)
w-115:45	2 (1, 2)	2 (1, 2)	−2 (−3, −2)	−3 (−3, −2)	0.62 (0.18, 2.61)
w-70:0	2 (1, 2)	2 (1, 2)	−3 (−3, −2)	−3 (−3, −2)	1.92 (0.58, 2.64)

wo, without-hand-cover; w, with-hand-cover; KI, kinesthetic illusion; Ext of KI, extension direction of KI; Flex of KI, flexion direction of KI; Agency, kinesthetic sense of agency; AVR, antagonist vibratory response; EMG, electromyography.

### 3.2 EMG activity for AVR (AVR-EMG activity)

**Figure 4:**
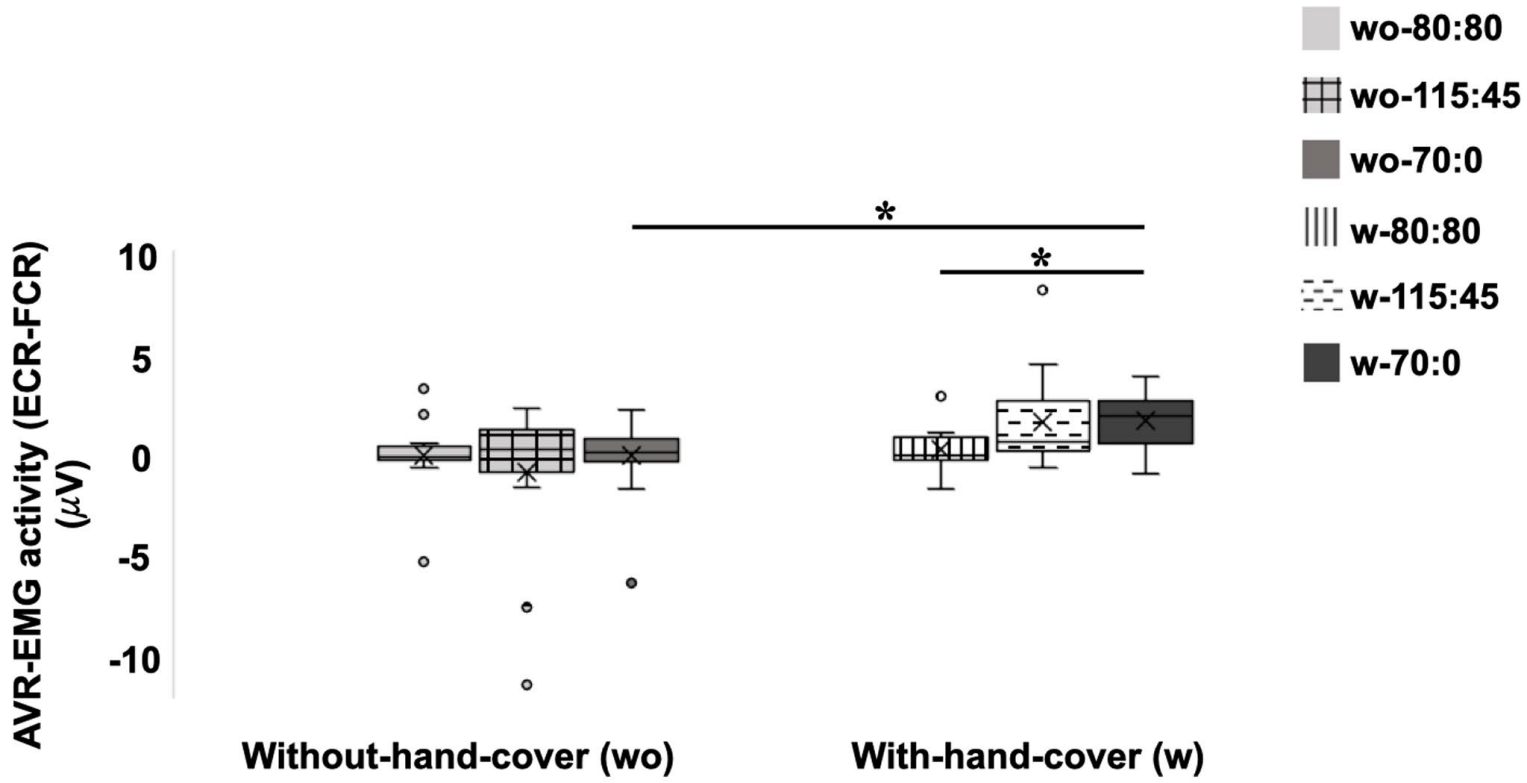
*Post hoc* test for AVR-EMG activity for all conditions. *Adjusted *p* < 0.05. ECR, extensor carpi radialis; FCR, flexor carpi radialis; AVR-EMG, antagonist vibratory response-electromyography.

Friedman’s test showed significant differences for all conditions of AVR-EMG activities (*p* < 0.001). Consequently, *post hoc* analysis revealed significant differences between the same frequency but different conditions: wo and w, w-70:0 vs. wo-70:0 (adjusted *p* = 0.027) and within the w-condition, w-70:0 vs. w-80:80 (adjusted *p* = 0.037) (Figure 4).

In addition, although no significant differences were observed between w-115:45 and either wo-115:45 or w-80:80 conditions, w-115:45 showed a descriptive increase in AVR-EMG activity compared to both wo-115:45 and w-80:80 (Table 1; Figure 4; Supplementary Figure S1).

### 3.3 ERD in specific frequency bands (*α*, *β*, and *γ*-ERD bands)

Friedman’s test across all six conditions showed significant main effects at α-C3 (𝑥^2^(5) = 12.37, Kendall’s *W* = 0.17, *p* = 0.03), β-P3 (𝑥^2^(5) = 17.32, Kendall’s *W* = 0.23, *p* = 0.004), and γ-P3 (𝑥^2^(5) = 11.11, Kendall’s *W* = 0.15, *p* = 0.049). However, *post-hoc* pairwise comparison did not reach significance after correction for multiple comparison. Time-frequency representation map (Figure 1b) demonstrated that ERD activation began shortly after stimulus onset and appeared to be sustained during the stimulation period (see Supplementary Figures S2–S4).

**Figure 5:**
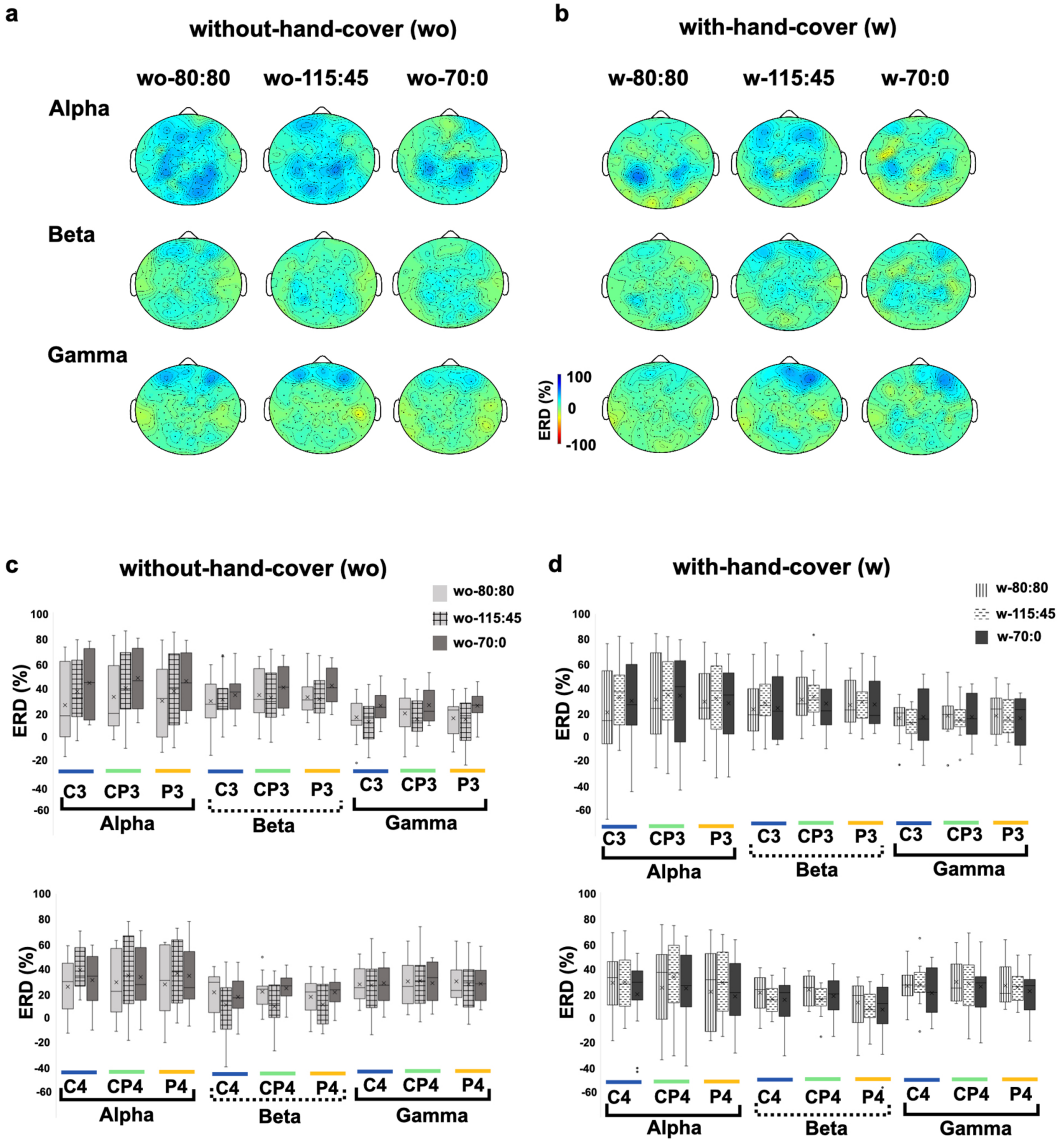
Spatial and spectral distribution of ERD across conditions. **(a,b)** Scalp topographic maps show the spatial distribution of ERD in the *α*, *β*, and *γ* frequency bands under wo and w conditions. **(c,d)** Boxplots display the ERD (%) distribution across ROIs (C3, CP3, P3; C4, CP4, P4) for each vibration condition. Boxes represent the interquartile range, horizontal lines indicate medians, and whiskers denote data variability. ERD, event-related desynchronization; *α*, alpha; *β*, beta; *γ*, gamma; wo, without-hand-cover; w, with-hand-cover; ROIs, regions of interest.

To provide a descriptive overview of ERD modulation across conditions, we illustrate overall patterns observed in the data. Both topographic maps (Figures 5a,b) and boxplots (Figures 5c,d) suggest a tendency for larger *α*-band ERD across ROIs. Because pairwise comparisons did not survive correction for multiple comparisons, these patterns are presented for visualization purposes only and should be interpreted cautiously. This tendency appeared spatially similar across left- and right-hemisphere ROIs; full details and additional visualizations are provided in Supplementary Table S1 and Supplementary Figures S2–S4.

In addition, the observation of the exploratory spectral assessment did not provide clear evidence that the analyzed EEG signals were dominated by vibration-related spectral peaks within the analyzed EEG frequency range.

### 3.4 Relationship between ERD bands and AVR-EMG activity: ERD bands and KI

The cortical and peripheral relationship was assessed specifically between cortical activity, as measured by ERD in specific frequency bands, and AVR-EMG activity in response to the direction of induced KI, representing peripheral muscle activity. Therefore, Spearman’s correlation was conducted under two conditions: (1) during distinct KI (combined w-115:45 and w-70:0 conditions) and (2) during less KI (combined wo-115:45 and wo-70:0 conditions).

The correlation analysis of the combined conditions of w-115:45 and w-70:0 showed a significant, weak correlation between α-C3-ERD and AVR-EMG activity (*ρ* = 0.37, *p* = 0.042) and between *γ*-C3-ERD and AVR-EMG activity (*ρ* = 0.37, *p* = 0.044) (Table 2). There was also a significant weak negative correlation between γ-CP3-ERD and KI according to the 7-point Likert scale (*ρ* = −0.36, *p* = 0.049) and a significant moderate negative correlation between γ-P3-ERD and KI according to the 7-point Likert scale (*ρ* = −0.40, *p* = 0.027) (Table 2). However, these significant correlations were not observed in the less KI: combined conditions of wo-115:45 and wo-70:0 in either ERD with AVR-EMG activity or ERD with KI (Table 2).

**Table 2:** ERD in specific frequency bands: correlations with AVR-EMG activity and KI.

ROIs	Parameters	Spearman’s correlation (*ρ*)			
		Distinct KI (w-115:45 and w-70:0)		Less KI (wo-115:45 and wo-70:0)	
		*ρ*	*p*-value	*ρ*	*p*-value
C3	α-ERD and AVR-EMG	0.37	0.042*	0.19	0.312
	β-ERD and AVR-EMG	0.31	0.091	0.08	0.639
	γ-ERD and AVR-EMG	0.37	0.044*	0.11	0.547
CP3	α-ERD and KI	−0.15	0.438	0.16	0.406
	β-ERD and KI	−0.12	0.516	0.06	0.756
	γ-ERD and KI	−0.36	0.049*	−0.12	0.530
P3	α-ERD and KI	−0.13	0.501	0.18	0.334
	β-ERD and KI	−0.08	0.657	0.14	0.450
	γ-ERD and KI	−0.40	0.027*	0.03	0.882

*Significant correlation (*p* < 0.05). w, with-hand-cover; wo, without-hand-cover; ERD, event-related desynchronization; α, alpha band; γ, gamma band; ROIs, region of interests; EMG, electromyography; AVR, antagonist vibratory response; KI, kinesthetic illusion.

## 4 Discussion

Our study integrated various sensory inputs; distinct KI were observed only under w-conditions (hidden limb context), but these effects were less apparent in wo-conditions (visible limb context). These findings align with the previous literature that the strength of KI is reduced when a direct conflict exists between visual signals and VIB-induced proprioceptive signals (Izumizaki et al., 2010; Proske and Gandevia, 2018). When the vibrated arm is hidden, the lack of task-relevant visual feedback allows the brain to prioritize on proprioceptive inputs. In this state, visual-kinesthetic conflict is minimized, leading to a distinct perception of movement (Calvin-Figuière et al., 2000; Izumizaki et al., 2010). Moreover, consistent with previous studies, we affirmed that KI was not induced when the frequency did not differ between the vibrated sides (Romaiguère et al., 2003; Taylor et al., 2017).

Analysis of AVR-EMG activity showed significant differences between the wo-70:0 vs. w-70:0 conditions and w-80:80 vs. w-70:0 conditions. These differences are congruent with established KI concept. In addition, no significant differences were observed between the wo-115:45 and w-115:45 pairs or w-80:80 and w-115:45 pairs; however, differences in AVR-EMG activity were observed. Several studies utilizing different designs have reported increased cortical excitability alongside AVR-EMG activity using Transcranial Magnetic Stimulation (TMS), EEG, and EMG (Calvin-Figuière et al., 2000; Romaiguère et al., 2003; Forner-Cordero et al., 2008; Naito et al., 2016). Accordingly, in line with KI and neuronal oscillation sensory processing concepts, our findings suggest that proprioceptive input may be prioritized during w-conditions. This reliance may be associated with altered sensorimotor processing in the absence of visual feedback regarding limb position (Calvin-Figuière et al., 2000).

Moreover, AVR-EMG activity showed no noticeable differences in the w-115:45 condition compared with the w-70:0 condition. This suggests that AVR-EMG activity may be more sensitive to frequency differences than to total frequency. One potential explanation is that sensitivity differences between conditions may have affected the results. In the w-115:45 condition, 115 Hz is likely to achieve a plateau response in muscle spindle tension with Ext of KI, while 45 Hz is contrary to a Flex of KI. Regardless of the ability of 115 Hz and 45 Hz to induce Ext of KI and Flex of KI, this could interfere with the induction of the illusion due to an increased cognitive load. Consequently, instead of amplifying the cortico-peripheral network, it elicited the same KI and Ext of KI responses as the w-70:0 condition. These findings highlight the potential importance of the perceived sensory integrations and their impact on the cortico-peripheral pathway and peripheral activity.

Regarding neuronal oscillation, the Friedman tests revealed significant main effects for ERD across conditions. However, no significant difference was observed in ERD bands between the wo and w conditions, despite KI was limited in the wo conditions. This pattern may suggest that ERD primarily reflect cortical engagement associated with vibration across experimental conditions rather than clear condition-specific differences. However, the relatively modest effect sizes suggest that more subtle ERD modulations may have fallen below the detectable threshold, particularly after Bonferroni adjustment for multiple comparison.

Building on these observations, one possible explanation for the observed ERD is that dual sensory inputs (tactile and visual inputs) modulate cortical activity, specifically across the sensorimotor and parietal scalp regions. This finding is consistent with previous observations of vibration and visual stimulation, which highlight sensorimotor and parietal cortex activation during KI (Bauer et al., 2009). In our study, the C3 and CP3 electrodes overlie the primary sensorimotor and somatosensory association areas, respectively; collectively, these sites reflect processing within sensorimotor network. In contrast, P3 electrode overlies the parietal cortex, a region commonly implicated in multisensory processing and integration near the somatosensory cortex particularly for touch, motion, and visual information (Scrivener and Reader, 2022; Raju and Tadi, 2025). Although no significant pairwise differences were observed between conditions, the modulation observed at P3 suggests sensitivity to the presence and combination of sensory inputs. Accordingly, ERD at P3 may be interpreted as reflecting neural processes associated with multisensory modulation of KI perception, rather than evidence of a specific localized computational mechanism.

The relationship between *α*-C3-ERD and AVR-EMG activity, as well as *γ*-C3-ERD and AVR-EMG activity, in combined w-115:45 and w-70:0 conditions, suggest that they were influenced by perceptual output intensity and EMG activity rather than ERD alone. The association observed at the C3 electrode may coordinated cortical and peripheral activity during VIB-induced KI and appears to be sensitive to specific VIB condition. Moreover, Shibata et al.’s observations, which revealed that α-band activity over the sensorimotor cortex reflects KI induced by VIS, our findings specifically show that this α-activity at C3 electrodes relates to muscle output (Shibata and Kaneko, 2019). This suggests a potential association where, within context of VIB, α-band involvement may be tied to the peripheral motor response accompanying the KI. However, the lack of significant findings for the *β* band in our study could be due to its specific function, which relates to motor execution, while vibration is a passively induced, sensory-driven movement without voluntary movement control (Başar et al., 2013).

Increasing evidence shows that *γ* is associated with hand movements and speed (Haverland et al., 2025). It also plays a vital role in perception, specifically regarding bottom-up activity following sensory input (Riddle et al., 2019). Furthermore, γ is essential for high-order cognitive function and movement related to parietal areas, where it helps strengthen interconnections between cortices (Riddle et al., 2019; Zhao et al., 2019; Guan et al., 2022; Haverland et al., 2025). Consistent with this role, our findings demonstrate that γ-oscillations, alongside the α band, are modulated during KI induced by VIB.

In this study, combined w-115:45 and w-70:0 conditions represent distinct KI, and we found a significant negative correlation between γ-ERD and KI at CP3 and P3 electrodes. This association suggests that γ-ERD in parietal association areas is modestly linked to the subjective intensity of the perceived KI. Rather than reflecting a definitive sensory gating mechanism (Buschman and Miller, 2007; Mancheva et al., 2017), this relationship may reflect partial involvement during multisensory processing of VIB-induced signals.

While previous studies (Mancheva et al., 2017; Lauzier et al., 2023) showed that cortical excitability and muscle responses co-occur during KI, our finding indicated γ-ERD is modestly associated with AVR-EMG activity. Specifically, γ-ERD over the primary sensorimotor cortex has been associated with motor output (Casini et al., 2006), while parietal γ-ERD has been associated with higher-order integration of sensory input (Hagura et al., 2007; Kanayama et al., 2012). These results suggest that γ-ERD in different regions may track distinct physical and perceptual aspects of the illusion. Importantly, these relationships are correlational and do not definitely establish causality or underlying specific neural mechanism. These modulations should be interpreted as reflecting relative changes in rhythmic synchrony within the experimental context, rather than as a direct measure of neural computation, information flow, or the efficiency of the KI.

These findings are consistent with the possibility that cortical-peripheral associations during distinct KI may involve descending motor pathways, which are generally known to interact with cortical systems in sensorimotor regulation (Chumin et al., 2022; Cruz et al., 2023). However, given the limitations of scalp EEG, we cannot directly assess subcortical contributions or distinguish between corticospinal and alternative descending systems. Therefore, this interpretation remains speculative and should be tested using multimodal approaches capable of directly examining cortico-subcortical interactions in future studies.

Furthermore, the relationship between ERD and AVR-EMG activities during the w-condition, particularly under distinctly KI conditions, requires careful interpretation. While not definitive, these results may imply that ERD may reflect a cortical state associated with sensorimotor processing. In contrast, EMG activity reflects the downstream motor-specific component that may accompany the subjective experience of the illusion. Therefore, these findings may suggest that changes in cortical activity are not necessarily accompanied by overt muscle activity, although further validation is needed. Future studies should employ methods such as corticomuscular coherence analysis to further clarify the time and frequency domain synchrony between ERD and EMG. This approach could help specify this relationship discreetly and predict whether ERD-EMG coupling predicts KI strength or not, as well as how sensory context modulates this relationship.

This study has several limitations. First, we focused on a healthy population, aged 18 to 35, which may limit the generalizability of the results across different age groups or neurological statuses. Second, although the *a priori* power analysis indicated that 19 participants would be required to detect medium-to-large effects, the final analyzable sample consisted of 15 participants. Consequently, the modest sample size reduces sensitivity to small effects and increases the risk of Type II error, particularly for cortical ERD response. Nevertheless, *post-hoc* sensitivity analysis indicated adequate detection of medium-to-large effects, whereas smaller effects may have remained undetected. Furthermore, although we applied Bonferroni-adjusted *post hoc* comparisons were applied to control for multiple testing, the large number of analyses across conditions, frequency bands, and electrode sites requires cautious interpretation. Accordingly, the present findings should be interpreted as exploratory effect-size observations rather than confirmatory hypothesis tests, and replication in a larger, adequately powered cohort will be necessary to establish the robustness and generalizability of these findings.

A further limitation is that the experimental conditions were presented in a fixed sequence rather than randomized or counterbalanced, which may have introduced order-related influences such as learning, sensory adaptation or fatigue despite the incorporation of rest intervals and the use of a standardized protocol; therefore, further studies should employ randomized or counterbalanced experimental designs for better control for potential order effects. Finally, our observation that *γ*-ERD data is related to both subjective feelings of KI and AVR-EMG activities in the absence of visual input remains speculative. The nature of the results cannot confirm causality and requires further confirmation using methods capable of directly assessing subcortical contributions. Nevertheless, this study provides initial insights into γ-ERD related to vibration with distinct KI. Future studies should further investigate the functional significance of γ-ERD within cortico-peripheral associations under varying sensory contexts.

## 5 Conclusion

This study demonstrates context-dependent associations between γ-band ERD, AVR-EMG activity, and subjective kinesthetic illusion during tendon vibration. These findings suggest condition- and region-specific cortical engagement accompanying perceptual and motor-related processes under distinct KI states. However, these relationships should be interpreted as correlational and preliminary, requiring further confirmation in larger and mechanistically targeted studies.

## Data Availability

The raw data supporting the conclusions of this article will be made available by the authors, without undue reservation.

## References

[ref1] Adham A. Bessaguet H. Struber L. Rimaud D. Ojardias E. Giraux P. (2024). Distinct and additive effects of visual and vibratory feedback for motor rehabilitation: an EEG study in healthy subjects. J. Neuroeng. Rehabil. 21:158. doi: 10.1186/s12984-024-01453-3, 39267092 PMC11391611

[ref2] Barone J. Rossiter H. E. (2021). Understanding the role of sensorimotor beta oscillations. Front. Syst. Neurosci. 15:655886. doi: 10.3389/fnsys.2021.655886, 34135739 PMC8200463

[ref3] Başar E. Başar-Eroğlu C. Güntekin B. Yener G. G. (2013). Brain’s alpha, beta, gamma, delta, and theta oscillations in neuropsychiatric diseases: proposal for biomarker strategies. Suppl. Clin. Neurophysiol. 62, 19–54. doi: 10.1016/b978-0-7020-5307-8.00002-8 24053030

[ref5] Bauer M. Oostenveld R. Fries P. (2009). Tactile stimulation accelerates behavioral responses to visual stimuli through enhancement of occipital gamma-band activity. Vis. Res. 49, 931–942. doi: 10.1016/j.visres.2009.03.014, 19324067

[ref6] Buschman T. J. Miller E. K. (2007). Top-down versus bottom-up control of attention in the prefrontal and posterior parietal cortices. Science 315, 1860–1862. doi: 10.1126/science.1138071, 17395832

[ref7] Calvin-Figuière S. Romaiguère P. Gilhodes J. C. Roll J. P. (1999). Antagonist motor responses correlate with kinesthetic illusions induced by tendon vibration. Exp. Brain Res. 124, 342–350. doi: 10.1007/s002210050631, 9989440

[ref8] Calvin-Figuière S. Romaiguère P. Roll J. P. (2000). Relations between the directions of vibration-induced kinesthetic illusions and the pattern of activation of antagonist muscles. Brain Res. 881, 128–138. doi: 10.1016/s0006-8993(00)02604-4, 11036150

[ref9] Casini L. Romaiguère P. Ducorps A. Schwartz D. Anton J. L. Roll J. P. (2006). Cortical correlates of illusory hand movement perception in humans: a MEG study. Brain Res. 1121, 200–206. doi: 10.1016/j.brainres.2006.08.124, 17020751

[ref10] Chumin E. J. Faskowitz J. Esfahlani F. Z. Jo Y. Merritt H. Tanner J. . (2022). Cortico-subcortical interactions in overlapping communities of edge functional connectivity. NeuroImage 250:118971. doi: 10.1016/j.neuroimage.2022.118971, 35131435 PMC9903436

[ref11] Cruz K. G. Leow Y. N. Le N. M. Adam E. Huda R. Sur M. (2023). Cortical-subcortical interactions in goal-directed behavior. Physiol. Rev. 103, 347–389. doi: 10.1152/physrev.00048.2021, 35771984 PMC9576171

[ref12] Forner-Cordero A. Steyvers M. Levin O. Alaerts K. Swinnen S. P. (2008). Changes in corticomotor excitability following prolonged muscle tendon vibration. Behav. Brain Res. 190, 41–49. doi: 10.1016/j.bbr.2008.02.019, 18378327

[ref13] Gilhodes J. C. Roll J. P. Tardy-Gervet M. F. (1986). Perceptual and motor effects of agonist-antagonist muscle vibration in man. Exp. Brain Res. 61, 395–402. doi: 10.1007/BF00239528, 3948946

[ref14] Goodwin G. M. McCloskey D. I. Matthews P. B. (1972). The contribution of muscle afferents to kinaesthesia shown by vibration induced illusions of movement and by the effects of paralysing joint afferents. Brain 95, 705–748. doi: 10.1093/brain/95.4.705, 4265060

[ref15] Guan A. Wang S. Huang A. Qiu C. Li Y. Li X. . (2022). The role of gamma oscillations in central nervous system diseases: mechanism and treatment. Front. Cell. Neurosci. 16:962957. doi: 10.3389/fncel.2022.962957, 35966207 PMC9374274

[ref16] Haegens S. Händel B. F. Jensen O. (2011). Top-down controlled alpha band activity in somatosensory areas determines behavioral performance in a discrimination task. J. Neurosci. 31, 5197–5204. doi: 10.1523/JNEUROSCI.5199-10.2011, 21471354 PMC6622699

[ref17] Hagura N. Takei T. Hirose S. Aramaki Y. Matsumura M. Sadato N. . (2007). Activity in the posterior parietal cortex mediates visual dominance over kinesthesia. J. Neurosci. 27, 7047–7053. doi: 10.1523/JNEUROSCI.0970-07.2007, 17596454 PMC6672236

[ref18] Harquel S. Cadic-Melchior A. Morishita T. Fleury L. Ceroni M. Menoud P. . (2025). Brain oscillatory modes as a proxy of stroke recovery. Neurorehabil. Neural Repair 39, 983–996. doi: 10.1177/15459683251363241, 41273103 PMC12686178

[ref19] Haverland B. Timmsen L. S. Wolf S. Stagg C. J. Frontzkowski L. Oostenveld R. . (2025). Human cortical high-gamma power scales with movement rate in healthy participants and stroke survivors. J. Physiol. 603, 873–893. doi: 10.1113/JP286873, 39786979 PMC11826070

[ref20] Izumizaki M. Tsuge M. Akai L. Proske U. Homma I. (2010). The illusion of changed position and movement from vibrating one arm is altered by vision or movement of the other arm. J. Physiol. 588, 2789–2800. doi: 10.1113/jphysiol.2010.192336, 20547672 PMC2956899

[ref21] Jensen O. Mazaheri A. (2010). Shaping functional architecture by oscillatory alpha activity: gating by inhibition. Front. Hum. Neurosci. 4:186. doi: 10.3389/fnhum.2010.00186, 21119777 PMC2990626

[ref22] Kanayama N. Tamè L. Ohira H. Pavani F. (2012). Top down influence on visuo-tactile interaction modulates neural oscillatory responses. NeuroImage 59, 3406–3417. doi: 10.1016/j.neuroimage.2011.11.076, 22173297

[ref23] Kaneko F. Takahashi R. Shibata E. (2017). 2-3-11. Pairing of kinesthetic illusion induced by visual stimulus and peripheral nerve stimulation causes sustained enhancement of corticospinal tract excitability. Clin. Neurophysiol. 128:e167. doi: 10.1016/j.clinph.2017.03.019

[ref24] Kito T. Hashimoto T. Yoneda T. Katamoto S. Naito E. (2006). Sensory processing during kinesthetic aftereffect following illusory hand movement elicited by tendon vibration. Brain Res. 1114, 75–84. doi: 10.1016/j.brainres.2006.07.062, 16920087

[ref25] Lauzier L. Perron M. P. Munger L. Bouchard É. Abboud J. Nougarou F. . (2023). Variation of corticospinal excitability during kinesthetic illusion induced by musculotendinous vibration. J. Neurophysiol. 130, 1118–1125. doi: 10.1152/jn.00069.2023, 37706230

[ref26] Le Franc S. Fleury M. Cogne M. Butet S. Barillot C. Lecuyer A. . (2020). Influence of virtual reality visual feedback on the illusion of movement induced by tendon vibration of wrist in healthy participants. PLoS One 15:e0242416. doi: 10.1371/journal.pone.0242416, 33216756 PMC7678999

[ref27] Leonardi G. Ciurleo R. Cucinotta F. Fonti B. Borzelli D. Costa L. . (2022). The role of brain oscillations in post-stroke motor recovery: an overview. Front. Syst. Neurosci. 16:947421. doi: 10.3389/fnsys.2022.947421, 35965998 PMC9373799

[ref28] Mancheva K. Rollnik J. D. Wolf W. Dengler R. Kossev A. (2017). Vibration-induced kinesthetic illusions and corticospinal excitability changes. J. Mot. Behav. 49, 299–305. doi: 10.1080/00222895.2016.1204263, 27588516

[ref29] Naito E. Morita T. Amemiya K. (2016). Body representations in the human brain revealed by kinesthetic illusions and their essential contributions to motor control and corporeal awareness. Neurosci. Res. 104, 16–30. doi: 10.1016/j.neures.2015.10.013, 26562333

[ref30] Okawada M. Kaneko F. Shindo K. Yoneta M. Sakai K. Okuyama K. . (2020). Kinesthetic illusion induced by visual stimulation influences sensorimotor event-related desynchronization in stroke patients with severe upper-limb paralysis: a pilot study. Restor. Neurol. Neurosci. 38, 455–465. doi: 10.3233/RNN-201030, 33325415

[ref31] Proske U. Gandevia S. C. (2018). Kinesthetic senses. Compr. Physiol. 8, 1157–1183. doi: 10.1002/cphy.c170036 29978899

[ref32] Raju H. Tadi P. (2025). Neuroanatomy, Somatosensory Cortex. Treasure Island, FL: StatPearls Publishing. Available online at: https://www.ncbi.nlm.nih.gov/books/NBK555915 (Accessed November 7, 2022). 32310375

[ref34] Riddle J. Hwang K. Cellier D. Dhanani S. D’esposito M. (2019). Causal evidence for the role of neuronal oscillations in top-down and bottom-up attention. J. Cogn. Neurosci. 31, 768–779. doi: 10.1162/jocn_a_01376, 30726180 PMC6701188

[ref35] Roll J. P. Vedel J. P. (1982). Kinaesthetic role of muscle afferents in man, studied by tendon vibration and microneurography. Exp. Brain Res. 47, 177–190. doi: 10.1007/BF00239377, 6214420

[ref36] Romaiguère P. Anton J. L. Roth M. Casini L. Roll J. P. (2003). Motor and parietal cortical areas both underlie kinaesthesia. Brain Res. Cogn. Brain Res. 16, 74–82. doi: 10.1016/s0926-6410(02)00221-5, 12589891

[ref37] Ryun S. Kim J. S. Lee H. Chung C. K. (2017). Tactile frequency-specific high-gamma activities in human primary and secondary somatosensory cortices. Sci. Rep. 7:15442. doi: 10.1038/s41598-017-15767-x, 29133909 PMC5684355

[ref38] Samantzis M. Wang C. Balbi M. (2025). Gamma oscillations and their role in orchestrating balance and communication following stroke. Neural Regen. Res. 20, 477–478. doi: 10.4103/NRR.NRR-D-24-00127, 38819055 PMC11317936

[ref39] Saur P. Hildebrandt J. Pfingsten M. Seeger D. Steinmetz U. Straub A. . (1996). Multidisciplinary treatment program for chronic low back pain, part 2. Somatic aspects. Schmerz 10, 237–253. doi: 10.1007/s004829600024 12799846

[ref40] Scrivener C. L. Reader A. T. (2022). Variability of EEG electrode positions and their underlying brain regions: visualizing gel artifacts from a simultaneous EEG-fMRI dataset. Brain Behav. 12:e2476. doi: 10.1002/brb3.2476, 35040596 PMC8865144

[ref41] Shibata E. Kaneko F. (2019). Event-related desynchronization possibly discriminates the kinesthetic illusion induced by visual stimulation from movement observation. Exp. Brain Res. 237, 3233–3240. doi: 10.1007/s00221-019-05665-1, 31630226

[ref42] Snyder D. B. Beardsley S. A. Hyngstrom A. S. Schmit B. D. (2023). Cortical effects of wrist tendon vibration during an arm tracking task in chronic stroke survivors: an EEG study. PLoS One 18:e0266586. doi: 10.1371/journal.pone.0266586, 38127998 PMC10735026

[ref44] Storch S. Samantzis M. Balbi M. (2021). Driving oscillatory dynamics: Neuromodulation for recovery after stroke. Front. Syst. Neurosci. 15:712664. doi: 10.3389/fnsys.2021.712664, 34366801 PMC8339272

[ref45] Taylor M. W. Taylor J. L. Seizova-Cajic T. (2017). Muscle vibration-induced illusions: review of contributing factors, taxonomy of illusions and user’s guide. Multisens. Res. 30, 25–63. doi: 10.1163/22134808-00002544

[ref46] Zhao M. Marino M. Samogin J. Swinnen S. P. Mantini D. (2019). Hand, foot and lip representations in primary sensorimotor cortex: a high-density electroencephalography study. Sci. Rep. 9:19464. doi: 10.1038/s41598-019-55369-3, 31857602 PMC6923477

