# Supplementary material for: Impact of sensory integration in modulating neuronal oscillation bands and antagonist vibratory response during co-vibration
Source: Front Hum Neurosci. 2026 Jul 29;20:1749961. doi: 10.3389/fnhum.2026.1749961 (PMC13462233; doi:10.3389/fnhum.2026.1749961)
Supplement: Supplementary file 1 [file Data_Sheet_1.PDF]

Supplementary Files

Supplementary Table 1: Median of α-ERD, β-ERD, and γ-ERD of C3, CP3, and P3 for all conditions

ROIs	Conditions	Median (IQR)		
		α-ERD [%]	β-ERD [%]	γ-ERD [%)]
C3	wo-80:80	16.53 (-0.42, 60.63)	25.82 (14.87, 42.01)	12.92 (8.87, 26.64)
	wo-115:45	31.06 (15.88, 61.47)	24.14 (21.58, 38.74)	15.73 (-2.17, 24.08)
	wo-70:0	43.26 (13.07, 70.85)	35.82 (22.05, 42.45)	23.45 (14.97, 33.08)
	w-80:80	13.60 (-5.18, 54.10)	18.44 (5.26, 39.60)	19.89 (9.42, 24.28)
	w-115:45	37.11 (10.23, 50.84)	25.71 (17.55, 43.22)	11.89 (3.34, 22.85)
	w-70:0	26.59 (9.90, 59.32)	21.35 (-1.50, 49.52)	14.86 (-2.39, 39.74)
CP3	wo-80:80	18.66 (8.47, 57.04)	29.94 (18.96, 54.57)	22.01 (7.65, 30.73)
	wo-115:45	37.3 (22, 67.7)	28.04 (15.37, 51.22)	12.38 (4.07, 28.70)
	wo-70:0	44.76 (22.16, 71.18)	39.71 (22.77, 56.31)	20.26 (12.45, 37.25)
	w-80:80	23.62 (2.92, 68.72)	27.34 (17.88, 48.96)	18.54 (6.88, 25.53)
	w-115:45	38.30 (14.29, 61.60)	30.55 (20.66, 42.46)	13.48 (8.87, 22.70)
	w-70:0	41.30 (-3.55, 62.31)	21.58 (10.21, 39.37)	15.21 (2.70, 35.97)
P3	wo-80:80	30.67 (-0.52, 54.17)	29.26 (21.16, 39.94)	21.13 (2.07, 23.96)
	wo-115:45	39.48 (9.17, 66.78)	30.23 (18.68, 45.01)	16.57 (-3.43, 26.93)
	wo-70:0	43.23 (20.69, 67.34)	39.19 (27.92, 55.2)	25.28 (18.95, 32.41)
	w-80:80	23.9 (14.82, 52.07)	22.43 (12.52, 46.45)	23.05 (2.57, 32.03)
	w-115:45	37.79 (6.75, 58.18)	29.91 (15.73, 37.14)	13.56 (3.84, 30.7)
	w-70:0	34.36 (2.9, 52.54)	17.94 (11.2, 45.85)	22.63 (-6.25, 31.41)

ROIs, region of interests; IQR, interquartile range; wo, without-hand-cover; w, with-hand-cover; ERD, event-related desynchronization; α, alpha band; β, beta band; γ, gamma band.

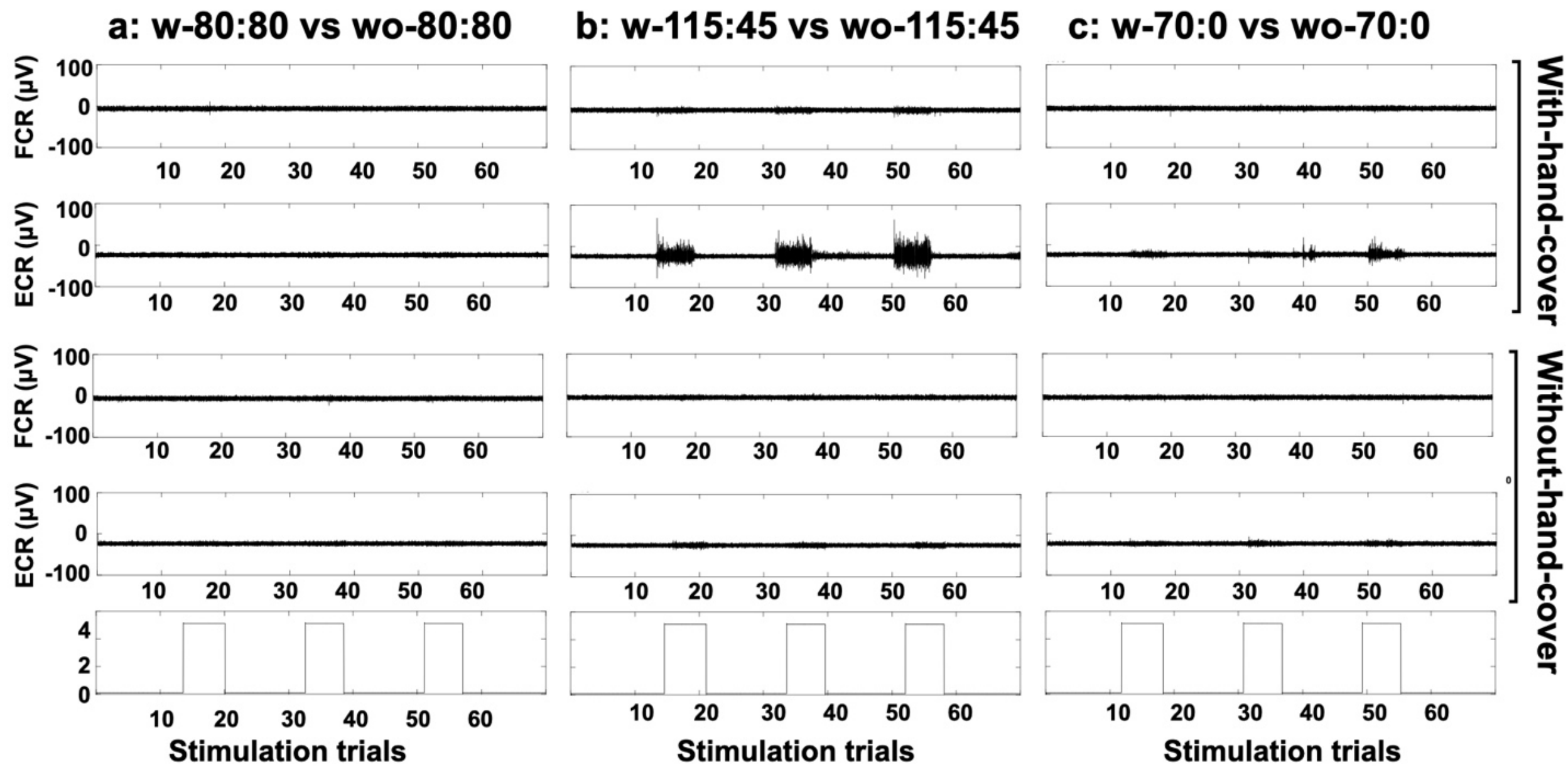

Supplementary Figure 1. One participant's raw data of AVR-EMG activities of all conditions between with-hand-cover (w) and without-hand-cover (wo) **a:** w-80:80 vs wo-80:80, **b:** w-115:45 vs wo-115:45, **c:** w-70:0 vs wo-70:0

Supplementary Figure 1: One participant's raw data of AVR-EMG activities of all conditions between with-hand-cover (w) and without-hand-cover (wo)

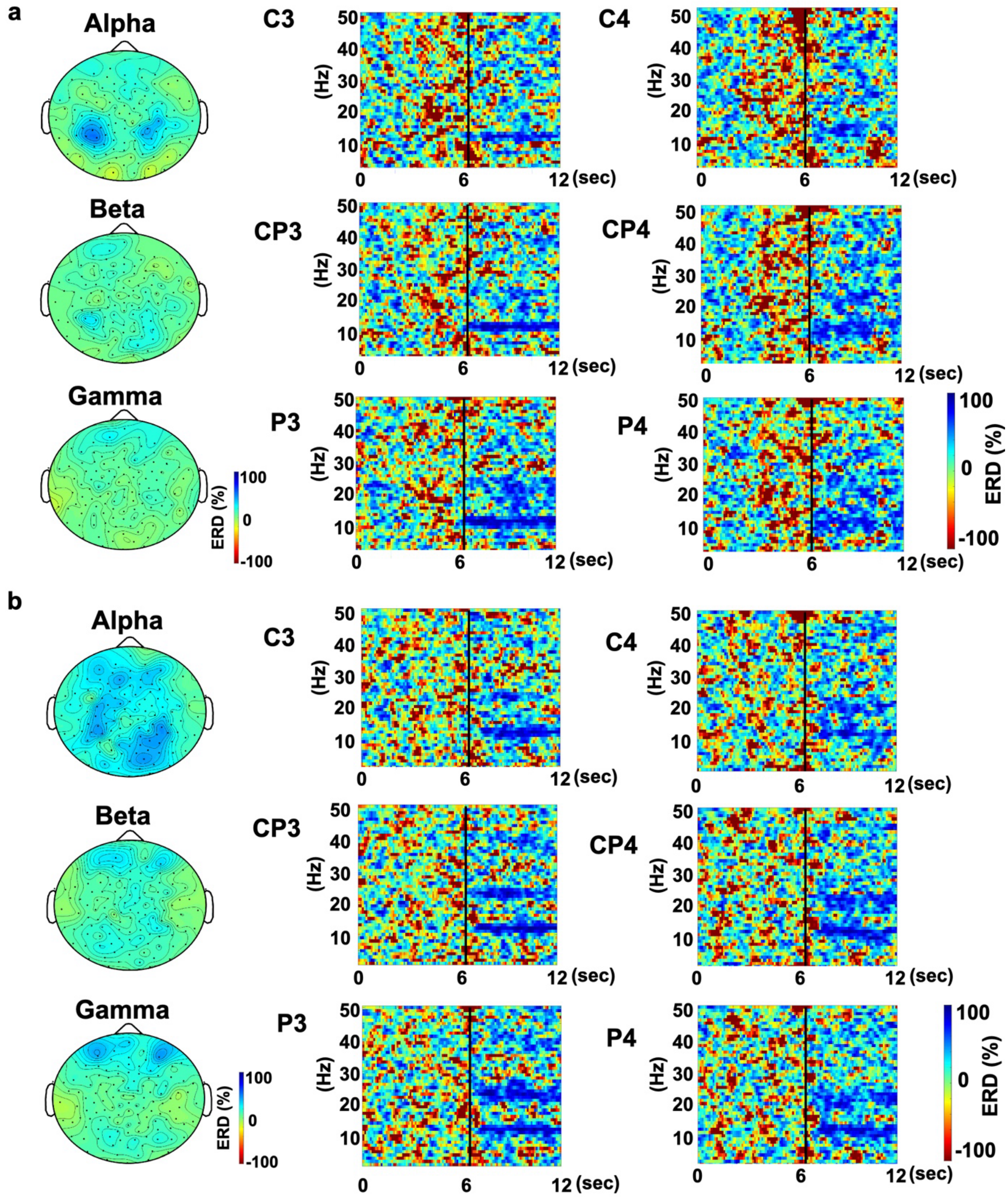

Supplementary Figure 2. One participant's ERD data in topographic map and time-frequency analysis of C3, CP3, P3, C4, CP4, P4 **a:** with-hand-cover of 80:80, **b:** without-hand-cover of 80:80

Supplementary Figure 2: One participant's ERD data in topographic map and time-frequency analysis of C3, CP3, P3, C4, CP4, P4

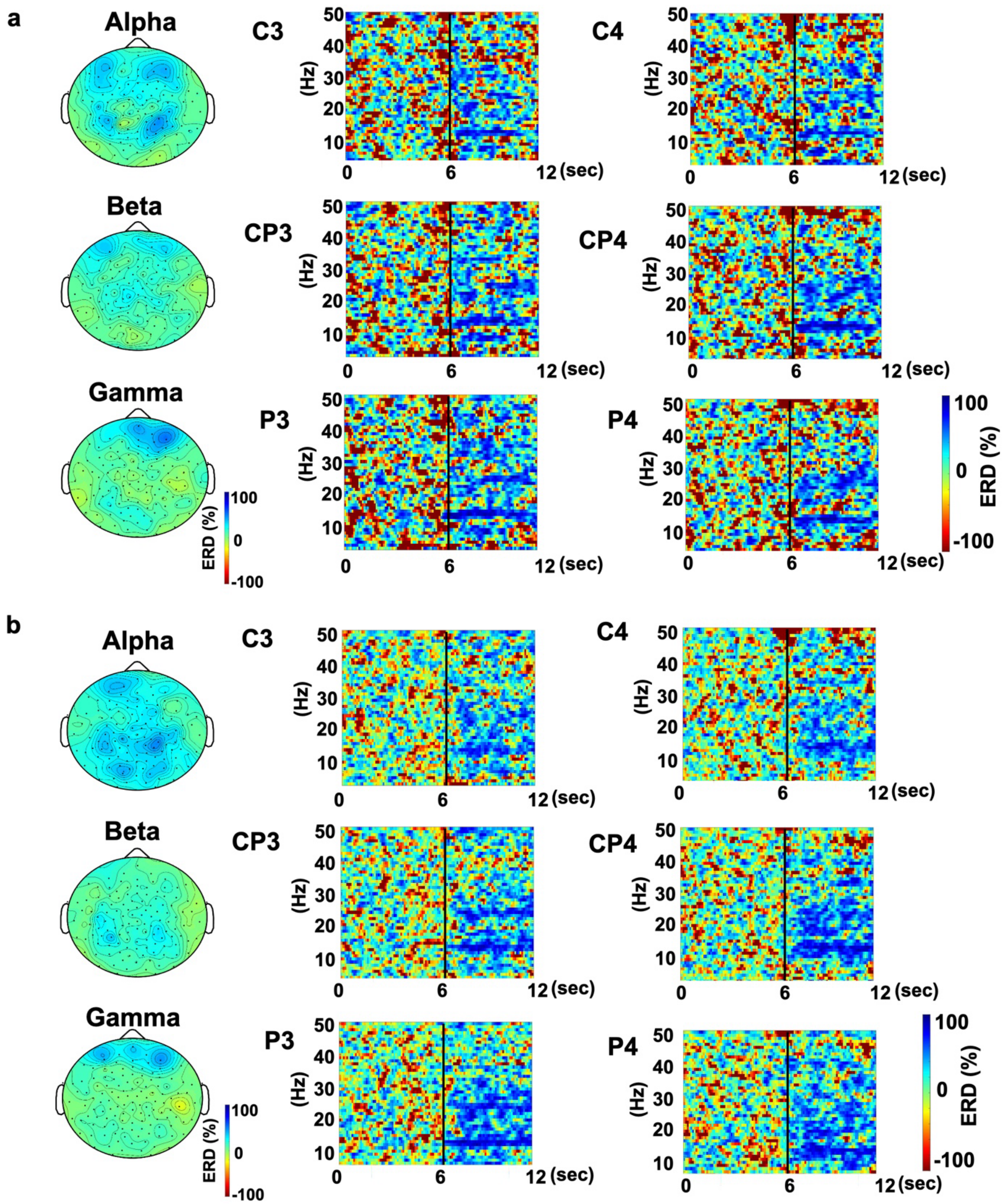

Supplementary Figure 3. One participant's ERD data in topographic map and time-frequency analysis of C3, CP3, P3, C4, CP4, P4 **a:** with-hand-cover of 115:45, **b:** without-hand-cover of 115:45

Supplementary Figure 3: One participant's ERD data in topographic map and time-frequency analysis of C3, CP3, P3, C4, CP4, P4

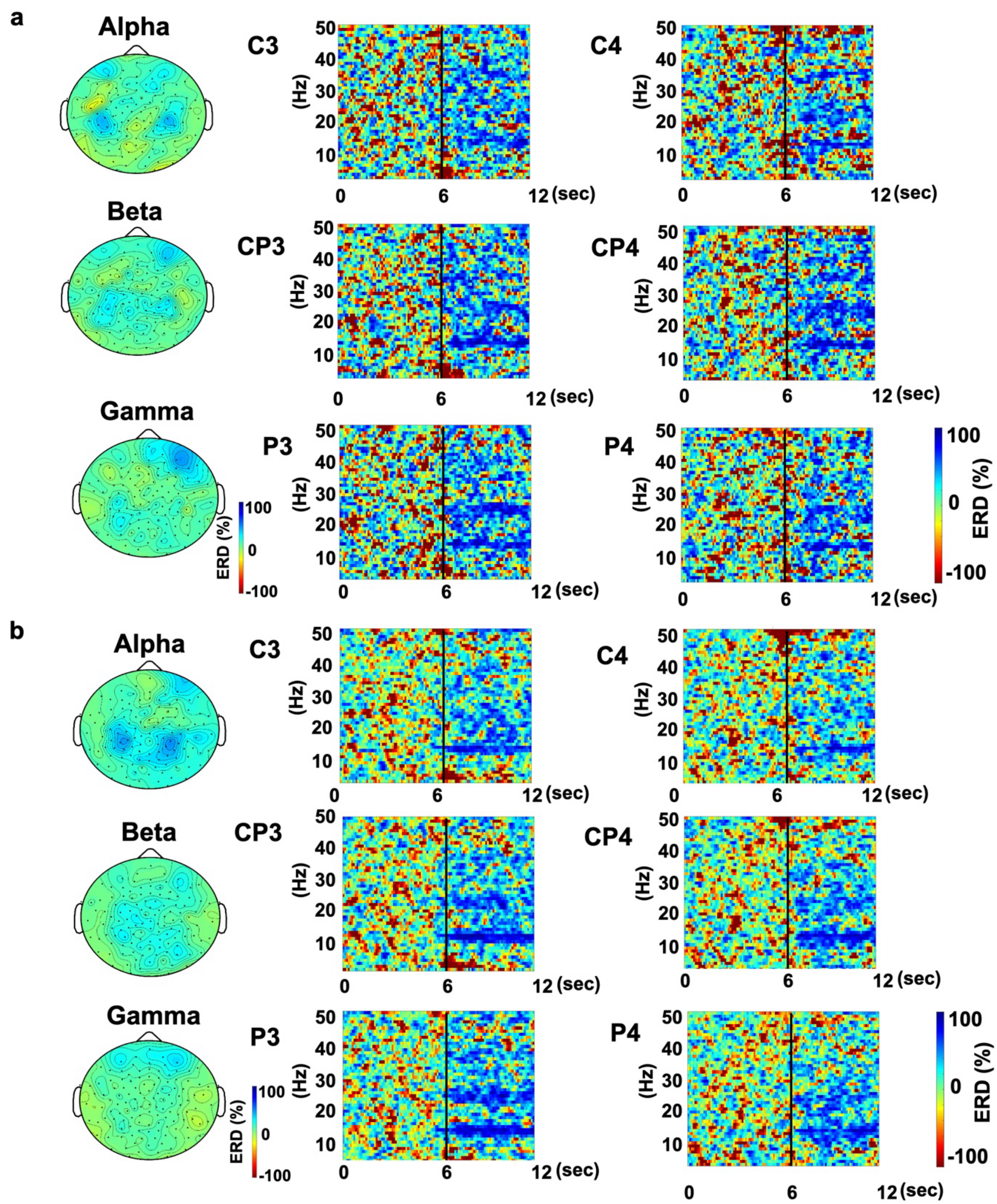

Supplementary Figure 4. One participant's ERD data in topographic map and time-frequency analysis of C3, CP3, P3, C4, CP4, P4 **a:** with-hand-cover of 70:0, **b:** without-hand-cover of 70:0

Supplementary Figure 4: One participant's ERD data in topographic map and time-frequency analysis of C3, CP3, P3, C4, CP4, P4
